# Supplementary material for: Promising Effects of Montelukast for Critically Ill Asthma Patients via a Reduction in Delirium
Source: Pharmaceuticals (Basel). 2024 Jan 18;17(1):125. doi: 10.3390/ph17010125 (PMC10819207; doi:10.3390/ph17010125)
Supplement: Supplementary file 1 [file pharmaceuticals-17-00125-s001.zip › pharmaceuticals-2785087-supplementary.pdf]

Supplementary Content

Table S1: Anti-asthma medications used to identify asthma patients

**Active pharmaceutical ingredients**
Beclomethasone, Levalbuterol, Ketotifen fumarate, Albuterol, Albuterol-sulfate, Albuterol-Ipratropium, Aminophylline, Arformoterol, Beclomethasone-Dipropionate, Fluticasone-Vilanterol, Arformeterol, Budesonide-formoterol, Mometasone-formoterol, Fluticasone furoate-vilanterol, Fluticasone propion-salmeterol, Fluticasone-Salmeterol, Formoterol, Formoterol-fumarate, Ipratropium-bromide, Ipratropium-Albuterol, Levalbuterol, Levalbuterol-Tartrate, Fometerol-Fumarate, Pirbuterol, Salmeterol, Salmeterol Xinafoate, Tiotropium Br/Olodaterol, Terbutaline Sulfate, Tiotropium-Bromide, Tiotropium-olodaterol, Ketotifen, and Zafirlukast.

Note：Listed here are the de-duplicated drug names, excluding the drug trade names as well as suffixes.

Table S2: Interaction Models of Exposure and Race

Variable	Coefficient	Standard Error	OR (95% CI)	p-value
Pre-ICU MTK exposure	β1：-0.527	0.219	0.591 (0.384-0.908)[c]	0.016[a]
Myocardial infarction	β2：-0.281	0.375	0.755 (0.362-1.574)	0.454[a]
Interaction item	β3：1.222	0.506	-	0.016[b]

Other covariates: race, age, gender, type of icu, SAPSII, SOFA, CCI, cerebrovascular disease, dementia, severe liver disease, and diabetes.

[a]A p-value less than 0.05 indicates a significant effect of the corresponding variable on the dependent variable (delirium), otherwise is no significant effect.

[b]The regression coefficient of the interaction term (β3) had a p-value <0.05 but the sign of β3 was opposite to that of β1, suggesting that myocardial infarction significantly attenuates the association between pre-ICU MTK exposure and delirium during hospitalization.

[c]In patients without myocardial infarction, the OR for Pre-ICU MTK exposure was 0.591 (i.e., Exp(β1)=0.591).In patients with myocardial infarction, the OR for Pre-ICU MTK exposure was 2.004 (i.e., Exp(β1+β3)=2.004).

Table S3: Interaction Models of Exposure and Myocardial Infarction

Variable	Coefficient	Standard Error	OR (95% CI)	p-value
Pre-ICU MTK exposure	β1：-1.438	0.507	0.237 (0.088-0.641)[c]	0.005[a]
Race(whites)	β2：-0.881	0.336	0.414 (0.214-0.801)	0.009[a]
Race(other)	β3：-0.871	0.450	0.419 (0.173-1.012)	0.053[a]
Interaction item(whites)	β4：1.284	0.566	-	0.023[b]
Interaction item(other)	β5：1.732	0.691	-	0.012[b]

Other covariates: age, gender, type of icu, SAPSII, SOFA, CCI, myocardial infarction, cerebrovascular disease, dementia, severe liver disease, and diabetes. Race was transformed into a dummy variable and black patients were used as a reference.

[a]A p-value less than 0.05 indicates a significant effect of the corresponding variable on the dependent variable (delirium), otherwise is no significant effect.

[b]The coefficients of the interaction terms (β4 and β5) had a p-value <0.05 and the signs of β4 and β5 were opposite to that of β1, suggesting that race significantly attenuates the association between pre-ICU MTK exposure and delirium during hospitalization.

[c]In black patients, the OR for Pre-ICU MTK exposure was 0.237 (i.e., Exp(β1)=0.237).For patients of white race and those of other races, the ORs of pre-ICU MTK exposure were 0.857 (i.e., Exp(β1+β4)=0.857) and 1.342 (i.e., Exp(β1+β5)=1.342) respectively.
